# Attitudes towards suicide in urban and rural China: a population based, cross-sectional study

**DOI:** 10.1186/s12888-016-0872-z

**Published:** 2016-05-26

**Authors:** Yaming Zou, Ricky Leung, Shao Lin, Mingan Yang, Tao Lu, Xianyun Li, Jing Gu, Chun Hao, Guanghui Dong, Yuantao Hao

**Affiliations:** Department of Medical Statistics and Epidemiology, School of Public Health, Sun Yat-sen University, 74 Zhongshan 2nd Road, Yuexiu District, Guangzhou, 510080 People’s Republic of China; Department of Health Policy and Management, School of Public Health, State University of New York at Albany, Albany, NY 12144-3445 USA; Department of Epidemiology and Biostatistics, School of Public Health, State University of New York at Albany, Albany, NY 12144-3445 USA; Department of Biostatistics and Epidemiology, Graduate School of Public Health, San Diego State University, San Diego, CA 92182 USA; Beijing Huilongguan Hospital, Beijing Suicide Research and Prevention Center, WHO Collaborating Center for Research and Training in Suicide Prevention, Beijing, 100096 China; Department of Preventive Medicine, School of Public Health, Sun Yat-sen University, 74 Zhongshan 2nd Road, Yuexiu District, Guangzhou, 510080 People’s Republic of China

**Keywords:** Attitudes towards suicide, Scale of Public Attitudes about Suicide (SPAS), Cross-sectional study, Residents, China

## Abstract

**Background:**

Suicide intervention programs have been guided by findings that attitude towards suicide and suicidal behavior may be causally linked. These findings also make it imperative to identify the factors that influence attitudes towards suicide. However, there has been little research on attitudes towards suicide among the general population, especially in low-income and middle-income countries. This population-based, cross-sectional study investigated the associated factors of attitudes towards suicide among a representative sample of urban and rural adult residents in China.

**Methods:**

A multi-stage, stratified random sampling approach was implemented to select participants. Data were collected by a survey using the Scale of Public Attitudes about Suicide (SPAS). The survey also collected some socio-demographic factors and suicidal history of participants. Statistical tests were conducted to identify associated factors that account for variations in attitudes towards suicide.

**Results:**

The residents in China generally hold a neutral attitude towards suicide. Attitudes towards suicide among Chinese residents were associated with age, duration of formal education, marital status, job and suicidal ideation. Different attitudinal subscales seemed not to share the same risk factors. However, gender, ethnicity, religious belief, housing style and economic status might not influence residents’ attitudes towards suicide. Attitudes towards suicide among Chinese urban and rural residents generally had no statistical difference with one notable exception: opinions on whether or not suicides and suicide attempts are different phenomena.

**Conclusions:**

Age, duration of formal education, marital status, job and suicidal ideation seem to have an impact on attitudes towards suicide among residents. Urban and rural residents have similar attitudes towards suicide with the only statistically significance difference being their opinions on whether or not suicides and suicide attempts are different phenomena.

**Electronic supplementary material:**

The online version of this article (doi:10.1186/s12888-016-0872-z) contains supplementary material, which is available to authorized users.

## Background

Suicide has posed a serious challenge to the world, especially in low-income and middle-income countries [1]. China has one of the highest suicide rates in the world. Its national suicide rate was 9.8 per 100,000 during the period from 2009–2011 [2]. Although recent research suggests that the overall suicide rate in China has decreased significantly, this trend may be unlikely to continue due to increasing stress levels and other socioeconomic conditions [3]. Thus, it is still important to identify factors that influence attitudes towards suicide in order to guide suicide prevention policy in China.

Early studies demonstrated that attitude towards suicide and suicidal behavior might be causally linked [4]. That is, attitudes towards suicide can lead to suicide in two ways: 1) attitudes towards suicide are able to accelerate the process from suicidal ideation to the eventual suicide (suicidal ideation, suicide plan, suicide preparation, suicide attempt and suicide) [5]; 2) a negative attitude towards suicide may decrease risk of suicide by prompting individuals to seek help [6]. These findings have guided intervention strategies and led to the development of community programs to reduce the number of suicides in rural and urban regions [7].

Most research on attitudes towards suicide has been conducted in high-income countries from a Western cultural perspective [8–10]. Although current studies have provided valuable insight into suicide, it is necessary to examine the applicability and generalizability of previous findings to non-Western cultures. For example, studies have found that religion is a significant predictor of positive attitudes toward suicide [11] and can be seen as a protective factor against suicide [12]. However, 91.6 percent of the Chinese population is Han ethnicity [13], of which only about 11 percent have religious beliefs or practices [14]. And for the few studies conducted in China, the measurement tool for attitudes towards suicide developed by Xiao et al. in 1999 used narrow measures that could not meet the requirements of the time [15, 16].

Attitudes towards suicide are affected by a number of factors including age, gender, religion, marital status, levels of education and prior contact with a suicidal peer. For example, older nurses were more likely to condemn suicide than their younger counterparts [17]; females were more likely to accept that suicide is preventable than males [18]. However, most research on attitudes towards suicide focused on a specific population group such as adolescents [8], college and high school students [19], social workers [20] and medical staff including psychiatrists [21], pharmacists [22] and nurses [17]. Unfortunately, there has been little research on attitudes towards suicide among the general public. For example, one study conducted by Renberg in Western countries had relatively low and different response rates (64 % in Sweden, 44 % in Norway and 92 % in Russia) and found that attitude towards suicide was affected by experience of suicidal behavior and self-reported suicidal expressions earlier in life [10]. However, this kind of research in low-income and middle-income countries such as China is even more lacking than in the West, making it difficult for these countries to build informed intervention programs.

The objective of this study was to examine the associated factors of attitudes towards suicide among urban and rural residents in China. The study collected a large sample in the city of Shenyang by applying a statistically validated, culture-specific scale. We assume that unique personal, social, psychological and environmental factors influence the general Chinese populations’ attitudes towards suicide. Therefore, a better understanding of these factors could guide suicide prevention strategies.

## Methods

### Sampling design

Between July 2008 and August 2009, a population-based, cross-sectional study was conducted in Shenyang, in northeast China. A study revealed that the average suicide rate in 1988-2000 among seven cities in Liaoning province was 7.11 per 100 thousand populations, which was almost equivalent to that of the same period of the whole country [23]. Shenyang, one of the largest cities in China, is comprised of urban and suburban districts and counties, with a total area of 13,308 km^2^ and a population of ten million in 2007. There were seven urban districts and five rural counties. Two communities were randomly selected from each urban district, and one village was randomly selected from each county. About 10 percent of families were then randomly selected from each of these communities or villages with only families who had resided in the community for at least three months during the past half-year included. Next, one participant (age ≥18 years old) was randomly selected from each household, without replacement. The procedures of this study were in accordance with ethical standards of the Institutional Review Board of Sun Yat-sen University. The ethics committee reviewed and approved the protocol of the study in compliance with the Declaration of Helsinki – Ethical Principles for Medical Research Involving Human Subjects.

### Data collection

All the data collection must be strictly carried out according to the above sampling design. All investigators received appropriate training by the lead researcher of this survey. First, residents of the identified households were introduced to the survey by members of the local neighborhood (or village) committee. If they agreed to participate, they were asked to sign a written informed consent. Then the investigators administered the survey in the participant’s home. Investigators read the items out for the participants then participants chose the most appropriate answer according to their own situation.

A total of 1255 individuals were randomly selected from 14 communities and eight villages and were invited to participate. A total of 983 (urban: 655; rural: 328) completed the survey with a response rate of 78.3 percent. Those who did not return a survey included 48 (3.8 %) who refused the survey, 17 (1.4 %) who had difficulty understanding the questionnaires, 21 (1.7 %) who provided inaccurate information (for example, carelessly read and randomly answered questions) and 186 (14.8 %) who were not home at the time.

### Measures

The Scale of Public Attitudes about Suicide (SPAS) (see Additional file 1) was used to assess attitudes towards suicide among residents in Shenyang. This scale was developed in China over three years with the express goal of capturing China-specific attitudes related to suicide. The efficacy of the SPAS scale has been validated by previous studies: the internal consistency and test-retest reliability of six of the seven SPAS subscales was good to excellent (Cronbach’s α and intraclass correlation coefficients [ICC] ranged from 0.62 to 0.87); but Subscale 6 (about the belief that suicide is an important social problem) had relatively low internal consistency (Cronbach’s α = 0.48) and fair test-retest reliability (ICC = 0.59) [24].

The SPAS includes 47 items; 44 items are divided into seven attitudinal subscales that reflect different attitudes towards suicide and the remaining three items assess basic knowledge about suicide. Jiao reported that these three knowledge-related items might not provide a comprehensive assessment of respondents’ knowledge about suicide [21]. So our study only utilized the 44 attitudinal items. The seven attitudinal subscales are as follows: Subscale 1 includes 6 items assessing beliefs about suicide prevention; Subscale 2 includes five items assessing beliefs about individuals’ ability to control suicidal tendencies; Subscale 3 includes ten items assessing respondents’ feelings of stigma towards suicide; Subscale 4 includes seven items assessing respondents’ feelings of empathy and understanding for persons with suicidal behavior; Subscale 5 includes six items assessing respondents’ beliefs about suicidal behavior as an effective method of controlling others; Subscale 6 includes five items assessing respondents’ views of suicide as a social problem; Subscale 7 includes five items assessing respondents’ categorization of suicides and suicide attempts.

Respondents to the 44 items in this survey were presented with a statement and asked to select their level of agreement from one of five choices: “definitely agree,” “mostly agree,” “neither agree nor disagree,” “mostly disagree,” and “definitely disagree.” In subscales 3, 4, 5 and 6, responses are scored from 5 to 1 (i.e., definitely agree = 5 to definitely disagree = 1); reverse scoring is used for subscales 1, 2 and 7 (i.e., definitely agree = 1 to definitely disagree = 5). The raw subscale scores are the sum of the item scores for the items in the subscales; these raw scores are converted to adjusted scores on a 0 to 100 scale using the following formula: (100*[raw subscale score-number of items in subscale]/[4*number of items in subscale]). The higher the adjusted subscale score, the more the respondent tends to agree with the attitude described in the name of the subscale. The seven adjusted subscale scores all range from 0 to 100 points, with higher scores (for example, more than 75) indicating greater agreement with the attitude described in the title of the subscale while low scores (for example, less than 25) indicating negative or contradictive attitude and middle scores (for example, around 25) indicating neutral attitude.

Socio-demographic conditions include gender (male/female), age (years), duration of education (years), ethnicity (Han/other ethnicity), religious belief (none coded as ‘no’; Catholicism, Christianity, Buddhism, Taoism, Islam and other religion coded as ‘yes’), marital status (divided into four categories: never married coded as ‘unmarried’; married, remarried, cohabiting and other marriage status coded as ‘married’; divorced and separated coded as ‘divorced’; widowed coded as ‘widowed’), insurance (self-supporting coded as ‘no’; cooperative medical service, free medical service, commercial insurance, medical insurance and other insurance coded as ‘yes’), job (unemployed and retired coded as ‘no’; all other occupations e.g. farmer, worker coded as ‘yes’), housing style (living alone coded as ‘yes’; dormitory, shared accommodation, living with family and other housing style coded as ‘no’), economic status (poor/general/good), region (urban/rural) and suicidal information including relatives’ suicidal behavior (the answer ‘0’ to the question “How many people committed suicide or suicide attempts among your parents, siblings, children and as such with blood relationship in the past?” coded as ‘no’ while ≥0 coded as ‘yes’) and respondents’ suicidal ideation (the answer to the question “Have you ever thought of suicide or intentionally hurting yourself in the past, whether or not you really did it?” divided into ‘yes’ and ‘no’) were also obtained for all survey respondents.

### Analysis

All the statistical analyses were conducted in SPSS 20.0. The Shapiro-Wilk test was used to examine whether or not each subscale score had a normal distribution. Mann-Whitney *U* test and Kruskal-Wallis H test were used to compare the mean scores of the seven subscales between two independent categorical variables and Spearman’s correlation coefficients were applied to test the significance when comparing the relationship between two continuous variables. Multiple linear regression analyses were used to identify independent factors associated with the seven attitudes towards suicide. Since age is universally considered strongly correlated with suicide-related behaviors, it was controlled for in all of the regression models. After forcing age into each of the regression models, all other variables were entered by a stepwise method if significant at the *p* < 0.05 level.

## Results

### Description of the seven subscale scores in the respondents

The seven subscale scores were not submitted to the hypothesis of normal distributions (Table 1). The median scores of each subscale ranged from 20.00 to 64.29. The scores of Subscale 4 (understanding and empathy) and Subscale 7 (dissimilarity) were 64.29 and 45.00 respectively, indicating residents were likely to feel empathy and understanding for persons with suicidal behavior and also to believe that suicides and suicide attempts are the same things. The scores of Subscale 3 (stigmatizing) and Subscale 6 (social importance) were 22.50 and 20.00 respectively, indicating residents did not hold stigmatizing attitudes about suicide and did not believe that suicide is an important social problem. The median scores of Subscale 1 (preventability), Subscale 2 (self-control) and Subscale 5 (controlling others) were about 50.00, indicating that the respondents held neutral attitudes regarding these aspects.

**Table 1:** The subscale scores of the Scale of Public Attitudes about Suicide (SPAS) among 983 residents in Shenyang

SPAS subscales	*N*	Test of normality		Median (Interquartile range)
		Shapiro-Wilk^a^	*P*	
^b^Subscale 1: preventability	983	0.990	<0.001	50.00 (29.17)
^b^Subscale 2: self-control	983	0.985	<0.001	50.00 (30.00)
^b^Subscale 3: stigmatizing	983	0.940	<0.001	22.50 (30.00)
^b^Subscale 4: understanding and empathy	983	0.980	<0.001	64.29 (25.00)
^b^Subscale 5: controlling others	983	0.991	<0.001	50.00 (29.17)
^b^Subscale 6: social importance	983	0.927	<0.001	20.00 (25.00)
^b^Subscale 7: dissimilarity	983	0.962	<0.001	45.00 (40.00)

^a^The Shapiro-Wilk test is used to assess the normality of the distribution of each subscale score. If *P* > 0.10 then the distribution is considered normal

^b^Each adjusted subscale score has a continuous range of 0–100

Subscale 1: Respondent believes suicide can be prevented

Subscale 2: Respondent believes individuals are able to control their own suicidal tendencies

Subscale 3: Respondent holds stigmatizing attitudes about suicide

Subscale 4: Respondent is understanding of and feels empathy for persons with suicidal behavior

Subscale 5: Respondent believes suicidal behavior is an effective method of controlling others

Subscale 6: Respondent believes that suicide is an important social problem

Subscale 7: Respondent believes that suicides and suicide attempts are essentially different

### Relationship between demographic, socioeconomic characteristics and SPAS subscales scores

As is shown in Table 2, younger residents were more likely to agree with all the subscales than elderly residents, but were less likely to feel empathy for persons with suicidal behavior. Residents with more educational background tended to agree with all the subscales but disagreed with Subscale 4 (understanding and empathy).

**Table 2:** The relationship between demographic, socioeconomic characteristics and each subscale score of the Scale of Public Attitudes about Suicide (SPAS) among 983 residents in Shenyang

Variables	*N*	^a^Subscale 1: preventability	^a^Subscale 2: self-control	^a^Subscale 3: stigmatizing	^a^Subscale 4: understanding and empathy	^a^Subscale 5: controlling others	^a^Subscale 6: social importance	^a^Subscale 7: dissimilarity
Age (years)								
* r*	983	−0.155^**^	−0.151^**^	−0.327^**^	0.074^*^	−0.162^**^	−0.206^**^	−0.246^**^
Years of education								
* r*	983	0.158 ^**^	0.156 ^**^	0.197 ^**^	−0.010	0.089^**^	0.055	0.084^**^
Median (Interquartile range)								
Gender								
Female	611	50.00 (29.17)	50.00 (30.00)	22.50 (30.00)^*^	64.29 (25.00)	50.00 (29.17)	20.00 (30.00)	45.00 (40.00)
Male	372	50.00 (29.17)	50.00 (35.00)	27.50 (32.50)	64.29 (28.57)	50.00 (29.17)	20.00 (30.00)	40.00 (40.00)
Ethnicity								
Han	897	50.00 (33.33)	50.00 (30.00)	22.50 (30.00)	67.86 (28.57)^*^	50.00 (29.17)	20.00 (25.00)	45.00 (40.00)
Other	86	58.33 (26.04)	50.00 (40.00)	27.50 (35.63)	60.71 (25.00)	50.00 (30.21)	20.00 (21.25)	45.00(45.00)
Religious belief								
Yes	147	50.00 (33.33)	50.00 (30.00)	25.00 (30.00)	64.29 (25.00)	52.08 (29.17)	20.00 (25.00)	40.00 (40.00)
No	836	50.00 (25.00)	22.50 (40.00)	20.00 (32.50)	67.86 (28.57)	50.00 (29.17)	20.00 (30.00)	50.00 (45.00)
Marital status^b^								
Unmarried	128	58.33 (32.29) ^**^	60.00 (28.75) ^**^	40.00 (20.00) ^**^	60.71 (21.43)^*^	62.50 (29.17)^**^	25.00 (23.75)^**^	55.00 (33.75)^**^
Married	731	50.00 (29.17)	50.00 (30.00)	22.50 (27.50)	64.29 (25.00)	50.00 (29.17)	20.00 (30.00)	40.00 (40.00)
Divorced	28	41.67 (32.29)	47.50 (52.50)	20.00 (43.75)	57.14 (39.29)	41.67 (28.13)	15.00 (33.75)	30.00 (52.50)
Widowed	96	50.00 (28.13)	45.00 (33.75)	16.25 (21.88)	67.86 (34.82)	50.00 (36.46)	10.00 (25.00)	35.00 (45.00)
Insurance								
Yes	590	50.00 (33.33)^*^	50.00 (35.00)	27.50 (37.50) ^**^	60.71 (30.36)^**^	50.00 (29.17)	20.00 (30.00)	45.00 (45.00)^**^
No	393	50.00 (29.17)	50.00 (30.00)	20.00 (27.50)	67.86 (28.57)	50.00 (29.17)	20.00 (30.00)	40.00 (40.00)
Job^b^								
Yes	538	54.17 (29.17) ^**^	55.00 (30.00) ^**^	27.50 (35.00) ^**^	64.29 (25.00)^**^	54.17 (25.00)^**^	25.00 (26.25)^**^	50.00 (41.25)^**^
No	148	43.75 (25.00)	45.00 (30.00)	25.00 (29.38)	60.71 (28.57)	50.00 (29.17)	20.00 (25.00)	45.00 (35.00)
Retired	296	50.00 (28.13)	50.00 (35.00)	17.50 (22.50)	71.43 (32.14)	50.00 (33.33)	15.00 (30.00)	25.00 (45.00)
Living alone								
Yes	80	50.00 (28.13)	50.00 (25.00)	20.00 (38.75)	67.86 (37.50)	50.00 (33.33)	20.00 (33.75)	40.00 (45.00)^*^
No	903	50.00 (29.17)	50.00 (30.00)	22.50 (30.00)	64.29 (25.00)	50.00 (29.17)	20.00 (25.00)	45.00 (40.00)
Economic status^b^								
Poor	236	45.83 (29.17)	50.00 (30.00)	25.00 (32.50)	60.71 (28.57)^*^	50.00 (29.17)	20.00 (30.00)	45.00 (40.00)
General	663	50.00 (29.17)	50.00 (35.00)	22.50 (30.00)	67.86 (28.57)	50.00 (29.17)	20.00 (25.00)	45.00 (40.00)
Good	84	45.83 (25.00)	40.00 (38.75)	20.00 (31.88)	62.50 (34.82)	50.00 (37.50)	20.00 (28.75)	35.00 (45.00)
Region								
Urban	655	50.00 (29.17)	50.00 (35.00)	22.50 (30.00)	64.29 (32.14)	50.00 (29.17)	20.00 (30.00)^**^	40.00 (40.00)^**^
Rural	328	50.00 (29.17)	50.00 (30.00)	25.00 (30.00)	64.29 (25.00)	54.17 (29.17)	25.00 (28.75)	50.00 (40.00)
Suicidal ideation								
Yes	57	50.00 (35.42)	50.00 (40.00)	37.50 (37.50) ^**^	57.14 (28.57)^**^	50.00 (27.08)	30.00 (22.50)^**^	50.00 (40.00)
No	917	50.00 (29.17)	50.00 (30.00)	22.50 (30.00)	67.86 (28.57)	50.00 (29.17)	20.00 (25.00)	45.00 (40.00)
Relatives’ suicidal behavior								
Yes	52	58.33 (29.17)^*^	50.00 (43.75)	27.50 (32.50)	60.71 (31.25)	50.00 (25.00)	20.00 (20.00)	45.00 (52.50)
No	922	50.00 (33.33)	50.00 (30.00)	22.50 (30.00)	67.86 (25.89)	50.00 (29.17)	20.00 (25.00)	45.00 (40.00)

^a^Each adjusted subscale score has a continuous range of 0–100

Subscale 1: Respondent believes suicide can be prevented

Subscale 2: Respondent believes individuals are able to control their own suicidal tendencies

Subscale 3: Respondent holds stigmatizing attitudes about suicide

Subscale 4: Respondent is understanding of and feels empathy for persons with suicidal behavior

Subscale 5: Respondent believes suicidal behavior is an effective method of controlling others

Subscale 6: Respondent believes that suicide is an important social problem

Subscale 7: Respondent believes that suicides and suicide attempts are essentially different

^b^multiple comparison test result: ^*^ *P* < 0.05; ^**^ *P* < 0.01

Compared with female residents, male residents tended to hold stigmatizing attitudes about suicide. Han people were more likely to feel empathy for persons with suicidal behavior. Unmarried residents were more likely to agree with all the subscales except for Subscale 4 (understanding and empathy) and Subscale 6 (social importance) than married residents. Compared with widowed residents, married residents tended to hold more stigmatizing attitudes about suicide and believe that suicide is an important social problem and suicides and suicide attempts are essentially different. The residents who had insurance tended to hold stigmatizing attitudes about suicide and believe that suicides and suicide attempts are essentially different but were less likely to feel empathy for persons with suicidal behavior. The residents who had jobs were likely to agree with all the subscales. The residents who were living with other people were more likely to believe that suicides and suicide attempts are essentially different than residents who were living alone. Residents of average or above average economic standing tended to feel empathy for persons with suicidal behavior. Rural residents tended to believe that suicide is an important social problem and that suicides and suicide attempts are essentially different. Residents who once had suicidal ideation were less likely to feel empathy for persons with suicidal behavior. Residents whose relatives once committed suicide tended to believe that suicide can be prevented. There were no significant differences based on the religious beliefs of residents.

### Factors associated with the seven subscales of attitudes towards suicide

Table 3 shows which factors may have influenced scores for each of the attitudinal subscales. For Subscale 1 (preventability), statistically significant factors included age, duration of formal education, insurance, job, relatives’ suicidal behavior and marital status; for Subscale 2 (self-control), statistically significant factors included duration of formal education, job and marital status; for Subscale 3 (stigmatizing), statistically significant factors included age, duration of education, suicidal ideation, job and marital status; for Subscale 4 (understanding and empathy), statistically significant factors included insurance, job and suicidal ideation; for Subscale 5 (controlling others), statistically significant factors included age, job and marital status; for Subscale 6 (social importance), statistically significant factors included age and suicidal ideation; for Subscale 7 (dissimilarity), statistically significant factors included age, region and marital status.

**Table 3:** Factors associated with the seven attitudes towards suicide assessed by the subscales of the Scale of Public Attitudes about Suicide (SPAS) among 983 residents in Shenyang^a^

SPAS subscales	*β*	Standard error	95 % CI of *β*	*P*
Subscale 1: Respondent believes suicide can be prevented				
*Constant*	41.845			
Age (years)	−0.143	0.072	(-0.284,-0.002)	0.047
Years of education	0.611	0.205	(0.208,1.014)	0.003
Insurance (Reference: no)	5.284	1.484	(2.371,8.196)	<0.001
Relatives’ suicidal behavior (Reference: no)	6.734	2.954	(0.936,12.532)	0.023
Marital status (Reference: unmarried)				
Married	−5.105	2.531	(-10.073,-0.138)	0.044
Divorced	−6.834	4.662	(-15.982,2.314)	0.143
Widowed	−1.981	3.751	(-9.342,5.380)	0.598
Job (Reference: no)				
Yes	6.383	1.958	(2.541,10.225)	0.001
Retired	4.101	2.551	(-0.904,9.107)	0.108
*R* ^*2*^	0.076			
Subscale 2: Respondent believes individuals are able to control their own suicidal tendencies				
*Constant*	48.764			
Age (years)	−0.190	0.065	(-0.318,-0.062)	0.004
Years of education	0.569	0.229	(0.119,1.019)	0.013
Job (Reference: no)				
Yes	5.812	2.190	(1.515,10.109)	0.008
Retired	6.789	2.756	(1.381,12.197)	0.014
*R* ^*2*^	0.036			
Subscale 3: Respondent holds stigmatizing attitudes about suicide				
*Constant*	55.946			
Age (years)	−0.269	0.053	(-0.373,-0.165)	<0.001
Years of education	0.634	0.191	(0.259,1.008)	0.024
Suicidal ideation (Reference: yes)	−8.717	2.739	(-14.092,-3.342)	0.549
Marital status (Reference: unmarried)				
Married	−5.374	2.372	(-10.082,-0.719)	0.221
Divorced	−2.604	4.341	(-11.123,5.915)	0.001
Widowed	−4.386	3.581	(-11.414,2.642)	0.002
*R* ^*2*^	0.121			
Subscale 4: Respondent is understanding of and feels empathy for persons with suicidal behavior				
*Constant*	43.016			
Age (years)	−0.028	0.052	(-0.130,0.075)	0.595
Insurance (Reference: no)	3.574	1.440	(0.747,6.401)	0.013
Suicidal ideation (Reference: yes)	7.438	2.803	(1.936,12.940)	0.008
Job (Reference: no)				
Yes	2.817	1.897	(-0.907,6.540)	0.138
Retired	5.860	2.384	(1.181,10.538)	0.014
*R* ^*2*^	0.029			
Subscale 5: Respondent believes suicidal behavior is an effective method of controlling others				
*Constant*	61.229			
Age (years)	−0.239	0.068	(-0.372,-0.106)	<0.001
Marital status (Reference: unmarried)				
Married	−1.527	2.532	(-6.496,3.442)	0.547
Divorced	−9.662	4.667	(-18.821,-0.503)	0.039
Widowed	−0.983	3.736	(-8.314,6.348)	0.793
Job (Reference: no)				
Yes	4.060	1.946	(0.241,7.880)	0.037
Retired	5.380	2.459	(0.556,10.205)	0.029
*R* ^*2*^	0.043			
Subscale 6: Respondent believes that suicide is an important social problem				
*Constant*	30.147			
Age (years)	−0.209	0.038	(-0.283,-0.134)	<0.001
Suicidal ideation (Reference: yes)	3.039	1.340	(0.409,5.669)	0.024
*R* ^*2*^	0.038			
Subscale 7: Respondent believes that suicides and suicide attempts are essentially different				
*Constant*	55.256			
Age (years)	−0.298	0.070	(-0.436,-0.160)	<0.001
Rural (Reference: urban)	5.378	1.853	(1.742,9.015)	0.004
Marital status (Reference: unmarried)				
Married	−6.326	3.242	(-12.688,0.036)	0.051
Divorced	−13.864	5.848	(-25.339,-2.388)	0.018
Widowed	−8.433	4.800	(-17.852,0.986)	0.079
*R* ^*2*^	0.073			

^a^The seven attitudes are assessed on continuous scales with a range of 0 to 100. In all seven analyses, the age was initially forced into the model and then all other variables were entered by a stepwise method if significant at the *p* < 0.05 level

## Discussion

As to our knowledge, this is the first study to examine the relationship between attitude towards suicide and personal and social factors among urban and rural residents in China. Our study found that Chinese residents’ attitudes towards suicide are generally affected by age, duration of formal education, marital status, job and suicidal ideation. It also appeared that different attitudinal subscales did not share the same risk factors. It would also appear that gender, ethnicity, religious belief, housing style and economic status might not influence residents’ attitudes about suicide.

Interestingly, we found the attitudes towards suicide among urban and rural residents in China to be the same except for the opinion as to whether or not suicides and suicide attempts are essentially different. Rural residents tended to believe suicides and suicide attempts are completely different, which is obviously a false belief ignoring the direct relationship between suicide attempts and suicides. Some researchers argue that suicides and medically serious suicide attempts actually are two overlapping populations sharing some common psychiatric factors and history features, for example, current mood disorder and previous suicide attempts [25]. Zhang even pointed out that suicides and medically serious attempters were of the same population in Chinese rural young adults [26]. Thus the government taking some measures to change this belief will help the early prevention of suicide.

Many prior studies in China and other countries report that attitudes towards suicide are related to personal characteristics like age, levels of education, occupation, marital status and other demographic factors [18, 21, 22, 27, 28]. In accordance with these prior findings, this current study also found differences in attitudes towards suicide between different subgroups of participants. Our result that younger residents showed negative or contradictive attitudes about suicide is parallel to Li’s research [28], which might be partly explained by the increasing intolerance to all forms of social deviance as one ages [28]. Our finding is consistent with prior reports [11, 27] that participants who have a higher educational level show positive attitudes towards suicide, so this relationship holds true for citizens of both Eastern and Western cultures. Our study also found that the unmarried or unemployed residents were more likely to hold negative attitudes towards suicide. It is easy to understand that unmarried and unemployment status may increase the vulnerability to negative consequences of stressful life events [29], and by this increases the feelings of loneliness, hopelessness, worries about the future and leads to reduced social support and quality of life [18].

Many studies [30] have found that religion serves as a protective factor against suicidal ideation or suicide. Unfortunately, our result has not demonstrated that religion is an influencing factor for attitude towards suicide. This might be explained by the fact that most residents in our study are Han ethnicity. China has 56 ethnic groups and Han is the largest ethnic population, comprising more than 91 % of the total population in China. Some of the minority population differ substantially from Han in terms of religious beliefs. Only about 11 % of Han ethnicity have religious beliefs or practices [14], while in some of the minority groups, religious beliefs and practices are more common. Thus the relationship between religion and attitude towards suicide might be very different from that in Han ethnic groups. We observed no difference in attitudes towards suicide by gender. We also found no association between living style and economic status and attitudes towards suicide. Some researchers suggest that higher household income protects against suicidal ideation [31], but this does not appear to extend to attitudes towards suicide. Other research also identified living along as a potential risk factor for suicide, but, again, based on our research this does not appear to extend to attitudes towards suicide in China [32].

The present study showed that residents who once had suicidal ideation held stigmatizing attitudes about suicide and felt less empathy for persons with suicidal behavior. We assume that stigmatizing and unsympathetic attitudes should increase the attractiveness of suicide when some unpleasant accidents occur. This implies that an advocate for the sympathy and compassion for suicide could help prevent suicidal behaviors. Our study also found that residents who have a history of suicide exposure tended to believe that suicide can be prevented. It might be due to the increased knowledge about suicide they could get as well as having confidence in suicide prevention.

Although several scales like Suicide Opinion Questionnaire, Suicide Attitude Questionnaire and Attitudes Toward Suicide have been widely used to determine the attitudes towards suicide, some controversies around their theoretical and methodological frameworks exist [33]. Furthermore, public attitude towards suicide is closely related to the region’s own cultural background, so directly applying scales that were developed abroad may be inappropriate. In China, the majority of existing scales measuring attitude towards suicide presently adopt Xiao’s Suicide Attitude Questionnaire. However, this questionnaire does not cover the aspects of public expectations, such as the view people hold about the preventability of suicide [15]. In order to comprehensively measure public attitudes towards suicide, we used the Scale of Public Attitudes about Suicide (SPAS), which was developed in China over a number of years.

Several limitations should be considered when interpreting our findings. First, the selected sample was representative of the adult (aged 18 years old or older) population and mainly China’s predominant Han population, thus the results may not generalize to the entire nation and minority groups. Second, the variables in the linear regression model might be not enough, so our models only explained a relatively small proportion of the variance in attitudes among residents. This is also a problem in other research, which highlights the need to find other important factors for future study. Lastly, because of its inherent limitations, cross-sectional study could not reveal the causal relationship between the factors and attitudes towards suicide or suicidal behaviors.

Despite these limitations, this study can still contribute to our understanding about the situation of attitudes towards suicide and associated factors among urban and rural residents in China. Like many other countries, China is increasingly aware of the importance of suicide prevention and interventions and is launching campaigns to address the issue. Through this study, more effective programs to reduce suicide rate could be developed and tested in the future.

## Conclusions

The residents in China generally hold a neutral attitude towards suicide. Attitude towards suicide among urban and rural residents in China had no difference except for the opinion whether suicides and suicide attempts are essentially different or not. Age, duration of formal education, marital status, job and suicidal ideation seem to have an impact on attitudes towards suicide among ordinary residents. Residents who are older, married, employed and with higher educational level tend to harbor positive attitudes towards suicide. Residents who once had suicidal ideation held stigmatizing attitudes about suicide and felt less empathy for persons with suicidal behavior. However, the generality and etiology of the relationships need to be addressed in more details in future research.

## Additional file

Additional file 1: Appendix 1. Scale of Public Attitudes about Suicide (SPAS) (translated from the original Chinese). (DOCX 16 Kb)

## Abbreviations

SPAS: scale of public attitudes about suicide
