# Supplementary material for: Attitudes towards suicide in urban and rural China: a population based, cross-sectional study
Source: BMC Psychiatry. 2016 May 26;16:162. doi: 10.1186/s12888-016-0872-z (PMC4881201; doi:10.1186/s12888-016-0872-z)
Supplement: Additional file 1: — Appendix 1. Scale of Public Attitudes about Suicide (SPAS) (translated from the original Chinese). (DOCX 16 Kb) [file 12888_2016_872_MOESM1_ESM.docx]

**Appendix 1 Scale of Public Attitudes about Suicide (SPAS)** (translated from the original Chinese)

| **Item sequence** | **Subscale 1: Respondent believes suicide can be prevented**  [Note: Items in this subscale are reverse coded; higher scores indicate disagreement with the content of the item] |
| --- | --- |
| 4 | Suicidal behavior is not predictable |
| 14 | In most situations, individuals and organizations should not intervene when a person is suicidal |
| 24 | Public mental health education does not help to decrease suicide rates |
| 34 | Suicide can’t be prevented |
| 37 | Better management of the means of suicide such as pesticides and lethal drugs will not reduce the occurrence of suicidal behavior |
| 43 | Once someone decides to take their life, it’s hard to change their mind |
| **Subscale 2: Respondent believes individuals are able to control their own suicidal tendencies**  [Note: Items in this subscale are reverse coded; higher scores indicate disagreement with the content of the item] | |
| 5 | Suicide occurs when an individual feels that there is no other choice |
| 15 | The factors that lead up to a suicide are beyond the control of the individual |
| 25 | Suicidal individuals are not responsible for taking the path that leads to suicide |
| 35 | Suicide is not due to the faults of the individual |
| 44 | Suicide is an act that is beyond the power of the individual to control |
| **Subscale 3: Respondent holds stigmatizing attitudes about suicide** | |
| 2 | Suicide is an act that betrays family and friends |
| 6 | Everyone looks down on the behavior of people who take their own lives |
| 11 | People are infuriated by suicidal behavior |
| 16 | Most people despise individuals who have suicidal behavior |
| 21 | A suicidal act is unquestionably an irresponsible behavior |
| 26 | People who die by suicide or attempt suicide are stupid |
| 31 | Suicidal behavior occurs in persons with low self-esteem |
| 36 | People with suicidal behavior are poorly educated |
| 41 | People with suicidal behavior are usually selfish |
| 47 | Suicide is an act of extreme cowardice |
| **Subscale 4: Respondent is understanding of and feels empathy for persons with suicidal behavior** | |
| 1 | In some circumstances suicide is a responsible act |
| 7 | Persons who are suicidal deserve our sympathy |
| 12 | Sometimes suicide is a courageous act |
| 17 | Taking one’s own life to benefit others is commendable |
| 22 | Most people pity those who die by suicide or attempt suicide |
| 32 | Sometimes suicide is a rational act |
| 40 | In some situations suicidal behavior is worthy of respect |
| **Subscale 5: Respondent believes suicidal behavior is an effective method of controlling others** | |
| 8 | People can use their death by suicide as a method to achieve other goals |
| 13 | The goal of most elders with suicidal behavior is to decrease the burden on the family |
| 18 | Most suicidal acts are primarily aimed at influencing others |
| 28 | The main goal of suicidal acts precipitated by interpersonal conflicts is to punish or threaten others |
| 38 | Making suicide threats is a way to alter the behavior of relatives and friends |
| 45 | Persons who attempt suicide can use their suicidal behavior to achieve personal goals |
| **Subscale 6: Respondent believes that suicide is an important social problem** | |
| 9 | Suicidal behavior causes substantial economic loss for the country |
| 23 | Suicidal behaviors often have negative effects on social stability |
| 29 | Suicide in the society negatively affects the healthy development of all adolescents |
| 39 | Suicide is a social problem that deserves the expenditure of substantial resources |
| 46 | Suicidal behavior has serious effects on the subsequent lives of family members |
| **Subscale 7: Respondent believes that suicides and suicide attempts are essentially different**  [Note: Items in this subscale are reverse coded; higher scores indicate disagreement with the content of the item] | |
| 3 | Persons who die by suicide and those who attempt suicide share the same personality traits |
| 19 | The severity of the life stressors experienced by persons who die by suicide is similar to that of persons who attempt suicide |
| 27 | The intent to die at the time of the suicidal act in persons who attempt suicide is as strong as that in those who die by suicide |
| 33 | The underlying causes of suicidal acts are the same in persons who die by suicide as the causes in persons who attempt suicide |
| 42 | The goals of the suicidal act in persons who die by suicide are similar to the goals of persons who attempt suicide |
| **Three questions about suicide-related knowledge** (not analyzed in this study) | |
| 10 | Persons who have attempted suicide may repeat their suicidal behavior |
| 20 | Talking about suicide-related issues with an individual does not precipitate suicidal behavior |
| 30 | People who say they intend to kill themselves may actually do it |
| Respondents to the 47 items in this survey (which are presented in the sequence shown in the ‘item sequence’ column) select the response that most closely represents their degree of agreement with the statement from five choices: ‘definitely agree,’ ‘mostly agree,’ ‘neither agree nor disagree,’ ‘mostly disagree,’ and ‘definitely disagree.’ In subscales 3, 4, 5, and 6 responses are scored from 5 to 1 (i.e., definitely agree=5 to definitely disagree=1); reverse scoring is used for subscales 1, 2, and 7 (i.e., definitely agree=1 to definitely disagree=5). The raw subscale scores are the sum of the item scores for the items in the subscales; these raw scores are converted to adjusted scores on a 0 to 100 scale using the following formula: (100*[raw subscale score-number of items in subscale]/[4*number of items in subscale]). The higher the adjusted subscale score, the more strongly the respondent agrees with the attitude described in the name of the subscale. | |
